# Avian influenza virus H9N2 infections in farmed minks

**DOI:** 10.1186/s12985-015-0411-4

**Published:** 2015-11-02

**Authors:** Chuanmei Zhang, Yang Xuan, Hu Shan, Haiyan Yang, Jianlin Wang, Ke Wang, Guimei Li, Jian Qiao

**Affiliations:** Department of Pathophysiology, College of Veterinary Medicine, China Agricultural University, Beijing, 100094 China; College of Animal Science and Veterinary Medicine, Qingdao Agricultural University, Qingdao, 266109 China

**Keywords:** Avian influenza virus, H9N2 subtype, Mink, Serology, Pathogenicity

## Abstract

**Background:**

The prevalence of avian H9N2 viruses throughout Asia, along with their demonstrated ability to infect mammals, puts them high on the list of influenza viruses with pandemic potential for humans. In this study, we investigated whether H9N2 viruses could infect farmed minks.

**Methods:**

First, we conducted a serological survey for avian influenza virus antibodies on a random sample of the field-trial population of farmed minks. Then we inoculated farmed minks with A/Chicken/Hebei/4/2008 H9N2 viruses and observed the potential pathogenicity of H9N2 virus and virus shedding in infected minks.

**Results:**

H9 influenza antibodies could be detected in most farmed minks with a higher seropositivity, which indicated that farmed minks had the high prevalence of exposure to H9 viruses. After infection, the minks displayed the slight clinical signs including lethargy and initial weight loss. The infected lungs showed the mild diffuse pneumonia with thickened alveolar walls and inflammatory cellular infiltration. Influenza virus detection showed that viruses were detected in the allantoic fluids inoculated supernatant of lung tissues at 3 and 7 days post-infection (dpi), and found in the nasal swabs of H9N2-infected minks at 3–11 dpi, suggesting that H9N2 viruses replicated in the respiratory organ, were then shed outwards. HI antibody test showed that antibody levels began to rise at 7 dpi.

**Conclusions:**

Our data provided the serological and experimental evidences that strongly suggested farmed minks under the natural state were susceptible to H9N2 viral infection and might be the H9N2 virus carriers. It is imperative to strengthen the H9N2 viral monitoring in farmed minks and pay urgent attention to prevent and control new influenza viruses pandemic prevalence.

## Introduction

Avian influenza virus (AIV) H9N2 have widely circulated in the world since its first detection from turkeys in 1966 [1]. The strong evidences suggested that H9N2 viruses had broken through species barriers and become capable of infecting various mammals, including humans [2–4]. Recent studies have also shown that H9N2 viruses are likely to contribute to the evolution of the H7N9 viruses that cause severe human respiratory infections in China. Sequencing analyses revealed that all the genes from these H7N9 viruses were of avian origin, with six internal genes from avian influenza H9N2 viruses [5]. The high prevalence of avian H9N2 viruses in poultry, along with their demonstrated ability to infect mammals, puts them high on the list of a candidate virus for triggering a possible influenza pandemic potential for humans, and emphasizes the urgency to the study of their ecology and pathogenicity in different poultries and animals [6].

Mink (*Mustela vison*), belonging to mustelidae, carnivora, mammalia, is a small fur-bearing animal with a high economic value. Recently, the scale of mink breeding in northern and eastern China has consistently increased, and the number of minks reached more than 60 million in 2012 [7]. As we know, ferrets as a model animal belonging to the family of *Mustelida*e, can become infected with avian influenza viruses including H9N2 viruses [8, 9]. There have been previous reports of minks being infected with influenza virus. Various investigators studied whether minks could be infected with influenza virus (including human-origin, swine-origin and also poultry-origin) and then transmit virus through physical contacts [10–12]. Cases describing naturally occurring viral infections and the clinical onset of symptoms have also been reported constantly [13–19]. Such as Li et al. [19] reported that farmed minks can become infected with H9N2 AIVs when housed under natural conditions in China. These reports provided evidence that minks are highly susceptible to influenza virus. In the present study, we conducted a serological survey to assess the prevalence of avian influenza virus exposure in farmed minks, and then observed the pathogenicity of H9N2 viruses and virus shedding in infected farm minks. Our data will offer further insight into the interaction between H9N2 influenza viruses and mammals.

## Materials and methods

### Virus

A/Chicken/Hebei/4/2008 (H9N2) (Ck/HB/4/08) (Genbank FJ499463–FJ499470), used in this study was isolated from chickens in northern China in 2008. Our prior detailed studies of its pathogenicity in mice revealed that infected mice exhibited high mortality rates and evidence of severe lung injury when inoculated with Ck/HB/4/08 virus without prior adaptation [20]. The virus was propagated in the allantoic cavities of 10-day-old embryonated SPF chicken eggs maintained at 37 °C for 72 h, which were then stored at −80 °C for use in later experiments. The 50 % egg infectious dose (EID_50_) was determined by serial titration of virus in 10-day-old embryonated SPF chicken eggs at 37 °C. The viral titers were calculated by the method of Reed and Muench as previously described [21, 22].

The referral antigens and positive serum used in this study were H5N1 (RE-5, clade 2.3.4) and H5N1 (RE-7, clade 7.2) antigen, H7N9 (A/Pigeon/Shanghai/S1069/2013) antigen, H9N2 (A/Chicken/Shanghai/10/01) antigen and their corresponding positive serum purchased from Harbin Veterinary Research Institute, Harbin, China, usually as a avian standard antigen and positive serum to detecting specific antibodies against avian influenza virus in China.

### Serological investigation

#### Blood sample collection and pretreatment

In order to assess the exposure of farmed minks to AIVs, during March to October 2013, 560 sera were collected from mink farms in five different area of Shandong province in China where more than 50 % of minks in the entire country are raised (Table 1). In our survey, all mink farms use raw poultry and poultry byproducts to feed minks and we could not find mink farms not using poultry as minks feed. In this study, 446 sera were collected from young mink showing no obvious signs of disease which had been born from the end of April to the beginning of May, and had been weaned at the end of July. Other 114 sera were collected from adults which were more than one year old and just gave birth (Table 3). Most of minks serologically examined were apparently healthy, no particular signs of any disease were observed, and most blood samples of minks were collected when slaughtered for fur. Young mink were not examined less than two months since they were too small to be bled. Blood samples of minks were collected from the tail or hind toes vein, then separated by centrifugation at 3,000 rpm for 15 min after clotting a little while, then transferred to new Eppendorf tubes, stored at −20 °C until tested for antibodies against influenza A virus. All serum samples were pretreated with receptor destroying enzyme (RDE, Denka Seiken Co. Japan) to remove nonspecific inhibitors before test.

**Table 1:** Summary of clinical mink samples examined in serological investigation

Age^a^	Number	Location	Collection date	Whether fed poultry products
Adult	42	Wendeng	March, 2013	Yes
Adult	28	Qingdao	April, 2013	Yes
Adult	40	Wendeng	April, 2013	Yes
Adult	4	Zhucheng	June,2013	Yes
Young	20	Zhucheng	June,2013	Yes
Young	8	Qingdao	June, 2013	Yes
Young	44	Zhucheng	July 2013	Yes
Young	4	Linyi	July, 2013	Yes
Young	110	Linyi	Aug, 2013	Yes
Young	114	Heze	Sep, 2013	Yes
Young	146	Heze	Oct, 2013	Yes

^a^Young: 2 to 6 months of age; Adult: 1 to 2 years of age

#### Hemagglutination Inhibition (HI) assays

Specific antibody titers against H5, H7 and H9 subtypes avian influenza virus were tested by hemagglutination inhibition (HI) assay using procedures described in the WHO Manual on Animal Influenza Diagnosis and Surveillance [23]. The referral antigens used in this study were H5N1 (RE-5) and H5N1 (RE-7) antigen, H7N9 (A/Pigeon/Shanghai/S1069/2013) antigen, H9N2 (A/chicken/Shanghai/10/01) and H9N2 (Ck/HB/4/08) antigen to detect specific antibodies against avian influenza virus. The antigens at a dilution were determined by HA to contain 4 HA units. 1 % specific pathogen free Guinea pig red blood cells were prepared. The HI titer was determined as the highest dilution of serum giving complete inhibition of haemagglutination. A HI titer ≥ 1:40 was considered to be positive. Three replicates were tested on each serum.

### Animal infection experiment

#### Experimental protocols

To assess the pathogenicity of H9N2 virus and virus shedding in infected farm minks, eight female 7-week-old minks were purchased from a mink farm located in Qingdao area, Shandong province. The infected minks and control minks were individually housed in the separately isolators and were seronegative (HI titer ≤1:10) for influenza A virus (determined by measuring hemagglutination inhibition titers against H9 virus as HI assays described). Six minks (infection group, named minks I1-6) were anesthetized by injection with xylazole hydrochloride, and inoculated intranasally (1 mL) with 10^7.5^EID_50_ of Ck/HB/4/08 H9N2 influenza virus. A negative control group (named minks C1-2) consisted of two minks which were inoculated with PBS as described above. All animal studies were conducted in compliance with established guidelines, and the study protocol was approved by the Animal Care Committee of China Agricultural University (Beijing, People’s Republic of China).

Firstly, all minks were monitored daily for clinical signs and symptoms such as lethargy, loss of appetite, cough, runny nose, shortness of breath, breathing difficulties, diarrhea, conjunctivitis, and death. Rectal temperature and body weight of mink C1, C2 and I5, I6 was measured and their blood samples were collected from the toes vein detecting the antibody titers every other day up to 15 days post-infection (dpi). Secondly, nasal washes and anal swabs were collected from all minks from 3 up to 15 dpi to observe the virus shedding. Nasal washes were collected by instilling 1 mL of PBS containing 1000 units/mL penicillin and 1000 μg/mL streptomycin into the nostrils of each mink, and the exudates were collected in a sterile Petri dish. Anal samples were collected by inserting a swab into the rectum, and then the swab was placed back into a sterile tube containing 1 mL PBS. The last part, on 3, 7, 11, and 15 dpi two minks (I1 and I2; I3 and I4; I5 and C1; I6 and C2) were euthanized respectively, collecting samples of heart, liver, spleen, lung, kidney, brain, trachea and intestine tissues (Table 4). Part of tissue was fixed in formalin for histopathological analysis, the other part of tissue was collected, homogenized and centrifuged, the supernatant was used for detecting the virus titration and tissue tropism in minks.

#### Histopathological analyses

Part of tissues collected from euthanized mink was fixed in 10 % neutralized phosphate-buffered formalin, then embedded in paraffin. Five-micrometer-thick sections were stained with hematoxylin-eosin for light microscopy.

#### Detection of H9N2 virus in tissues

Tissues collected were weighed and homogenized in 1 mL cold PBS containing 1000 units/mL penicillin and 1000 μg/mL streptomycin. Following centrifugation of the homogenates, aliquots of the supernatants were inoculated into three 10 day-old SPF embryonated chicken eggs. Thereafter, the allantoic fluids of the embryos were tested using the hemagglutination (HA) assay using chicken RBCs. HA titers ≥ 2^4^ was considered to be positive. All the HA positive samples and the allantoic fluids of the embryos which died during the incubation were confirmed further using the RT-PCR analysis. Primers (P1:ACGCTTACCCTATTCAAGACGC; P2:TAGTCCTGACCAACCTCCCTCT) were designed based on the HA gene conserved sequence of H9N2 AIVs shown in GenBank, and synthesized by TAKARA Biotechnology (DALIAN) Co., Ltd. (Japan). Viral RNA was extracted from the allantoic fluid using Trizol reagent following the manufacturer’s protocol (Invitrogen Co., LTD; Grand Island, NY, USA). cDNA was synthesized by M-MLV reverse transcription and amplified by PCR.

#### Detection of H9N2 virus in nasal washes and anal swabs

Total RNA was extracted from all samples using Trizol reagent. The presence of virus was detected by fluorogenic quantitative RT-PCR using an AIV- H9 RT-PCR Assay Kit (QIAGEN; Limberg, Austria). RT-PCR was conducted in a 20 μL solution containing 15 μL reaction buffer, 0.25 μL Taq enzyme, 0.25 μL of RT-PCR enzyme, and 10 μL of RNA. The reaction conditions were: 1 cycle at 42 °C for 30 min; 1 cycle at 92 °C for 3 min; followed by 5 cycles at 92 °C for 10 s, 45 °C for 30 s and 72 °C for 1 min, and 40 cycles at 92 °C for 10 s and 60 °C for 30 s. *Ct* number ≤30.0 was considered to be positive. Negative results *Ct* number showed ‘None’.

#### Detection of antibody titers by HA/HI assay

Serum samples from mink C1,C2 and I5,I6 collected were tested H9-specific antibody titers 1–15 dpi by HI assay using procedures as HI assays described. The antigen used was homologous viruses at a dilution determined by HA to contain 4 HA units.

## Results

### Serological investigation results

By the HI assay, 45.4 % (254/560) of serum samples tested were positive against Ck/HB/4/08 (H9N2). 47.5 % (266/560) of serum samples tested are positive against A/Chicken/Shanghai/10/01 (H9N2). 10 (1.8 %) and 36 (6.4 %) serum samples tested are positive for H5N1 (RE-5) and H5N1 (RE-7) respectively. No antibodies were found against H7 subtype viruses (Table 2). This results showed that H5 and H9 subtype influenza virus were prevalent in farmed minks, however, no H7 subtype influenza virus existed in minks. And the rates of serum positivity against two H9 antigens were very high in both young and adult minks (Table 3).

**Table 2:** Seroprevalence of avian influenza virus in farmed minks

Viral antigens	Antibody titers using HI test^a^							% (No. of positive samples/total no.)
	≤1:20	1:40	1:80	1:160	1:320	1:640	1:1280	
H5N1(RE-5)	550	8	2	0	0	0	0	1.8 % (10/560)
H5N1(RE-7)	524	36	0	0	0	0	0	6.4 % (36/560)
H9N2 (A/chicken/Shanghai/10/01)	294	64	80	74	30	16	2	47.5 % (266/560)
H9N2(A/Chicken/Hebei/4/2008)	306	80	76	55	26	15	2	45.4 % (254/560)
H7N9(A/Pigeon/Shanghai/S1069/2013)	560	0	0	0	0	0	0	0(0/560)

^a^The HI titer was expressed as the reciprocal of the highest serum dilution that completely inhibited hemagglutination of 4 HA units of the virus. HI titers ≥ 1:40 was considered to be positive

**Table 3:** Distribution of antibodies against AIVs in young and adult minks^a^

Age	% virus seroprevalence (Number of positive samples/total number)				
	H5N1 (RE-5)	H5N1 (RE-7)	H9N2 (A/chicken/Shanghai/10/01)	H9N2 (A/Chicken/Hebei/4/2008)	H7N9 (A/Pigeon/Shanghai/S1069/13)
Young	2.2 % (10/446)	6.7 % (30/446)	48.0 % (214/446)	43.9 % (196/446)	0 (0/446)
Adult	0 (0/114)	5.3 % (6/114)	45.6 % (52/114)	50.8 % (58/114)	0 (0/114)

^a^Young: 2 to 6 months of age; Adult: 1 to 2 years of age

### Animal study results

#### Clinical signs

**Fig. 1:**
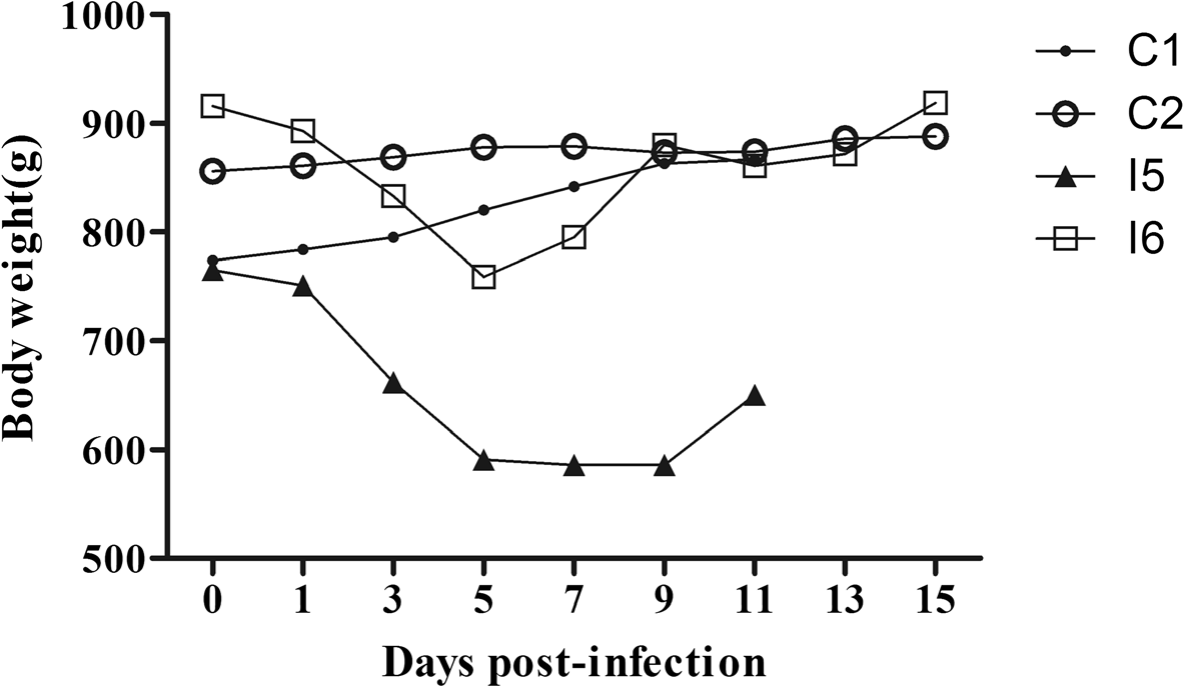
Mink body weights. Minks in infected group (I5 and I6) displayed an initial weight loss, their weights rose again at 5–9 post-inoculation day. No weight loss in the control minks (C1 and C2)

Following inoculation, the minks displayed symptoms which included lethargy and a dry nose. During the post-inoculation period, the body temperatures of infected minks remained stable, and was similar to the control minks. While infected minks I5 and I6 displayed an initial weight loss, their weights rose again on 5–9 dpi. The minks C1 and C2 in the control group showed no evidence of weight loss (Fig. 1). No animal died during the study, and the control minks inoculated with PBS behaved normally, and showed no suspicious clinical signs.

#### Histopathological lesions

**Fig. 2:**
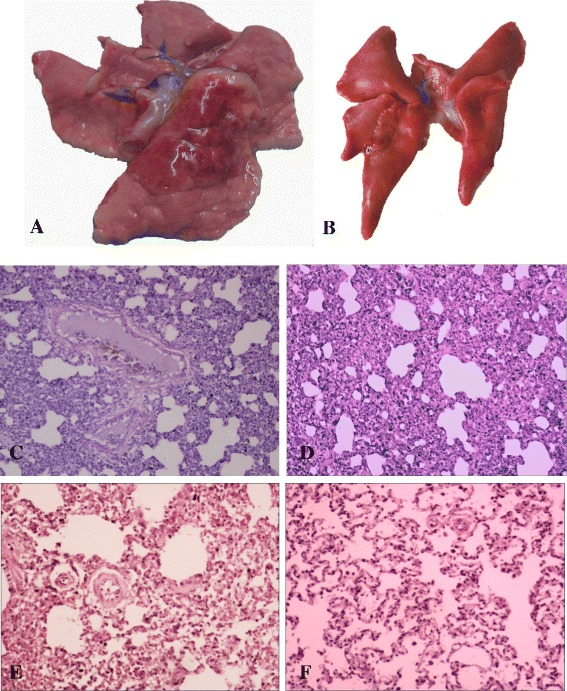
Gross pathology and histopathological changes. **a** Lung of infected mink showed areas of extravasated blood and partial consolidation at 3 dpi. **b** No significant changes in the lung of control mink. Histopathology with hematoxylin-eosin staining in infected lung is shown in C–F (H&E, ×40). **c** On 3dpi, alveolar walls of minks in the infection group were markedly thickened and contained much of exudates resulting from inflammation. Substantial amounts of serous fluid had seeped out of veins, and infiltration of inflammatory cells into lung tissue was observed. **d** On 7 dpi, part of alveolar fusion and part of alveolar consolidated with exudates were observed. **e** On 11 dpi, the injury of the lung became to ease, but still have some exudes in the alveolar and some inflammatory cells infiltrated. **f** By 15 dpi, only a little of inflammatory cells infiltrated in alveolar

Compared to the control minks, more obvious pathological changes were observed in lung tissue samples taken from the infected minks with H9N2 virus, showing areas of congestion, edema and compensatory emphysema (Fig. 2a); H&E staining revealed that on 3 dpi, alveolar walls of the infected minks were markedly thickened and contained much of exudates resulting from inflammation. Substantial amounts of serous fluid had seeped out of veins, and infiltration of inflammatory cells into lung tissues were observed (Fig. 2c). On 7 dpi, part of alveolar fusion and part of alveolar consolidated with exudates were observed (Fig. 2d). On 11 dpi, the injury of the lung became to ease, but still had some exudes in the alveolar and some inflammatory cells infiltrated (Fig. 2e). By 15 dpi, only a little of inflammatory cells infiltrated in alveolar (Fig. 2f). Minks in the control group showed no pathological changes in the lungs (Fig. 2b).

#### Virus tissue tropism in minks

Tissue samples obtained from all minks at 3, 7, 11 and 15 dpi, respectively, were tested for the presence of virus using virus isolation procedures with 10-day-old SPF chicken eggs (Table 4). Allantoic fluid inoculated by tissues of minks was analyzed by HA and RT-PCR. Virus replication was observed in the lung tissues of mink I1,I2 and I4 on 3 and 7 dpi respectively. Interestingly, virus replication was observed in the heart, brain, kidney and lung of mink I2 at 3 dpi. However, virus was not detected in the other tissues. In contrast, no virus was detected from control minks. These results showed that H9N2 replication mainly occurred in the respiratory tract. These result is validated and consistent with RT-PCR analyses.

**Table 4:** Distribution of H9N2 influenza viruses in experiment minks^a^

		Euthanized post-infection	Lung	Brain	Heart	Kidney	Spleen	Intestine	Trachea	Liver
Infection group	I1	3	+	-	-	-	-	-	-	-
	I2	3	+	+	+	+	-	-	-	-
	I3	7	-	-	-	-	-	-	-	-
	I4	7	+	-	-	-	-	-	-	-
	I5	11	-	-	-	-	-	-	-	-
	I6	15	-	-	-	-	-	-	-	-
Control group	C1	11	-	-	-	-	-	-	-	-
	C2	15	-	-	-	-	-	-	-	-

^a^Results showed that allantoic fluids inoculated supernatant of tissues were detected by HA and RT-PCT. HA titers ≥ 2^4^ was considered to be positive (+). “-” indicates negative. Results of HA was validated and consistent with RT-PCR

#### Virus shedding

Nasal washes and anal swabs were collected from the minks in both groups from 3 to 15 dpi, and analyzed using an AIV-H9 RT-PCR Assay Kit (QIAGEN). H9N2 virus could be detected in the nasal washes of infected minks at 3–7 dpi, and in the nasal washes of mink I5 at 9 and 11 dpi (Table 5). No virus was found in the nasal washes of control minks. No virus was detected in all anal swabs. These results indicated that infected minks shed virus from the upper respiratory tract.

**Table 5:** Detection of H9N2 influenza viruses in nasal washes^a^

		Days post-infection						
		3	5	7	9	11	13	15
Control group	C1	None	None	None	None	None	/	/
	C2	None	None	None	None	None	None	None
Infection group	I1	+	/	/	/	/	/	/
	I2	+	/	/	/	/	/	/
	I3	+	+	+	/	/	/	/
	I4	+	+	+	/	/	/	/
	I5	+	+	+	+	+	/	/
	I6	+	+	+	None	None	None	None

^a^ “+” indicates positive. “None” indicates negative. “/” indicates it has not be tested, because this mink has been euthanized before

#### Antibody response in experiment minks

**Fig. 3:**
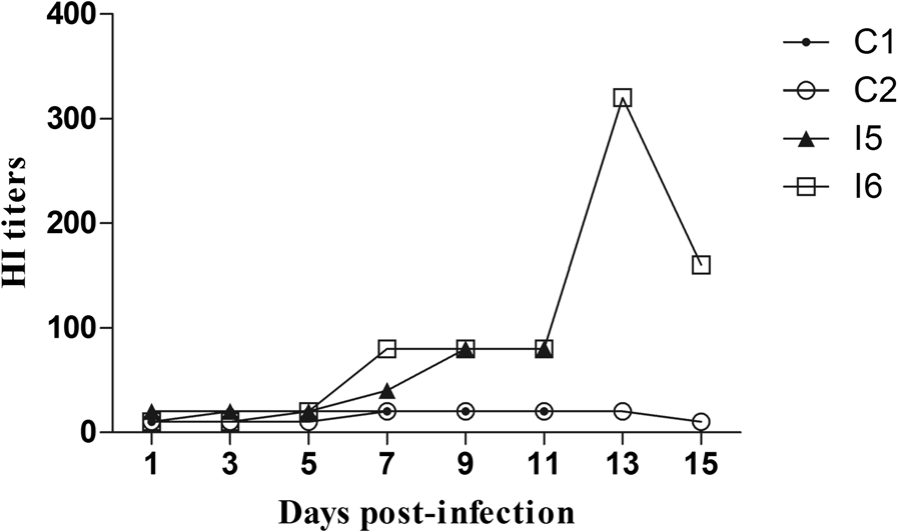
HI titers on different days post-infection. Antibody levels of infected minks I5 and I6 began to increase at 7 dpi, persisted until 15 dpi. HI titers of control minks C1 and C2 did not change significantly

HI results showed that antibody levels of infected mink I5 and I6 began to increase on 7 dpi, and persisted until 15 dpi. However, antibody titers of control minks C1 and C2 did not change significantly (Fig. 3). These serology data demonstrated that H9N2 replicates in minks, and induce production of anti-H9 antibodies in infected animals.

## Discussion

H9N2 viruses have circulated in domestic poultry in mainland China since 1994, and also been detected in different species of wild birds [24]. A study by Li et al. [25] showed that six H9N2 viruses found in live poultry markets in southern China could be transmitted in ferrets by respiratory droplet. Thus the widespread dissemination of H9N2 viruses poses a threat, because such droplets can function as “vehicles” for delivering different influenza virus subtypes from avian species to mammals, and H9N2 virus become capable of infecting various mammals [26, 27]. As the economic market for furs has risen in recent years, the number of mink farms in China has dramatically increased. Minks are obligate carnivores and have a high demand for high-quality protein. In China, owing to their lower costs, raw poultry and poultry byproducts, including heads, bones, viscera and blood are widely used to feed fur animals after they have been mixed with other ingredients [7, 28]. Therefore, under this condition, it is fully possible that H9N2 viruses could infect minks, and farmed minks might become the carriers of viruses. According to our survey for farmers, diseases with symptoms similar to those of a common cold infect animals in mink farms in the late winter, early spring, and during rainy summers; however, such infections are rarely fatal and thus do not receive much attention by farmers. Lowly pathogenic avian influenza (H9N2) has no obvious clinical symptoms, reduce the possibility of finding timely these cases, providing the virus with adaptive mutations occur in minks. Therefore, under this condition, it is fully possible that AIVs could infect minks and transmit in the minks. To explain how the cross-species contamination arises, Gagnon et al. [13] have reported maybe because minks are fed with uncooked pork by-products and non-processed, raw remains of swine coming from slaughterhouse facilities (i.e., discarded tissues, such as lung, which is the target of swine influenza virus). Yoon et al. [14] also believe it is reasonable to speculate that the source of FLUAV might have been uncooked turkey meat, due to the common occurrence of cross-species transmission of SIV to turkeys. Another reason probably of minks infected with influenza virus, farmed mink are kept outdoors in rows of cages covered only by a roof providing shade and shelter. The feed, consisting of uncooked chicken or fish by-products mixed with cereals, is administered on top of each cage and is thus freely accessible for birds perching on the netting. As described by L. Englund [29], it is well known that mink farms are frequently invaded by sparrows and other birds which forage for food and contaminate mink farms with faecal droppings. This situation is a common occurrence on mink farms in China.

In this study, we report for the first time the seroprevalence of avian influenza viruses in mink populations. Our results showed that antibodies against two H9N2 viruses were found in numerous mink plants, and the rates of serum positivity against more pandemic strains, [e.g., A/chicken/Shanghai/10/01 (H9N2) (47.5 %)] were higher than those against H9N2 [(Ck/HB/4/08) (45.4 %)], suggesting that the H9N2 virus has already been prevailed among the farmed mink population of China. In animal studies, we observed the potential pathogenicity of H9N2 virus and virus shedding in infected minks. After infection, the minks displayed the slight clinical signs, lungs had lesions, virus replicated in the lung tissues and could be eliminated outwards by nasal fluid. The titers of H9-specific antibody began to rise in sera collected at 7 dpi, and continued to increase up to 15 dpi. These results, such as their disease course and body reactions, and nonfatal infection are similar to the ferrets after infected by H9N2 virus [8, 9].

We provide serological and experimental evidence that strongly suggests farmed minks are susceptible to H9N2 virus infection, strongly suggesting that feeding raw poultry meat is a risk factor for cross-species transmission of influenza A virus from avian to farmed raised small mammals like minks, which could lead to eventual adaption of avian origin new influenza A virus to mammals, and reminding us to pay more attention to detecting its onset in minks. It is very important to do epidemiological surveillance of influenza virus not only within common susceptible animals, like avian populations, but also for all other species where intensive production and high geographic densities of animals may favor the appearance of new influenza virus isolates. Furthermore, through monitoring AIVs in other animal can not only prevent and control new influenza viruses pandemic prevalence, and provide more information for people’s public health.
